# CrustyBase v.2.0: new features and enhanced utilities to support open science

**DOI:** 10.1186/s12864-024-10033-7

**Published:** 2024-01-29

**Authors:** Cameron J. Hyde, Tomer Ventura

**Affiliations:** 1 Queensland Cyber Infrastructure Foundation, The University of Queensland, Level 5 Axon Building, St. Lucia, QLD 4072 Australia; 2 Centre for Bioinnovation, University of the Sunshine Coast, Sippy Downs, QLD 4556 Australia

**Keywords:** Crustacea, Differential gene expression, Online resource, RNA-Seq, Transcriptome

## Abstract

**Background:**

Transcriptomes present a rich, multi-dimensional subset of genomics data. They provide broad insights into genetic sequence, and more significantly gene expression, across biological samples. This technology is frequently employed for describing the genetic response to experimental conditions and has created vast libraries of datasets which shed light on gene function across different tissues, diseases, diets and developmental stages in many species. However, public accessibility of these data is impeded by a lack of suitable software interfaces and databases with which to locate and analyse them.

**Body:**

Here we present an update on the status of CrustyBase.org, an online resource for analysing and sharing crustacean transcriptome datasets. Since its release in October 2020, the resource has provided many thousands of transcriptome sequences and expression profiles to its users and received 19 new dataset imports from researchers across the globe. In this article we discuss user analytics which point towards the utilization of this resource. The architecture of the application has proven robust with over 99.5% uptime and effective reporting of bugs through both user engagement and the error logging mechanism.

We also introduce several new features that have been developed as part of a new release of CrustyBase.org. Two significant features are described in detail, which allow users to navigate through transcripts directly by submission of transcript identifiers, and then more broadly by searching for encoded protein domains by keyword. The latter is a novel and experimental feature, and grants users the ability to curate gene families from any dataset hosted on CrustyBase in a matter of minutes. We present case studies to demonstrate the utility of these features.

**Conclusion:**

Community engagement with this resource has been very positive, and we hope that improvements to the service will further enable the research of users of the platform. Web-based platforms such as CrustyBase have many potential applications across life science domains, including the health sector, which are yet to be realised. This leads to a wider discussion around the role of web-based resources in facilitating an open and collaborative research community.

**Supplementary Information:**

The online version contains supplementary material available at 10.1186/s12864-024-10033-7.

## Background

Over the last decade, reduced costs and improved efficiency of RNAseq technologies has resulted in an enormous surge in the production of transcriptomic data in life science research [1, 2]. These data can be used to obtain genetic sequences of coding DNA region, but more importantly they enable accurate quantitation of mRNA abundance across a set of samples in the given experiment. In this way, transcriptomics is quite different to conventional genomics, which is almost entirely dedicated to the study of sequence variation between individuals, populations or species. While a genome or genetic sequence can generally be extracted from any individual with the appropriate genotype, transcriptomics is typically more concerned with variation in biological context between samples, and the associated flux in mRNA abundance across expressed genes. It should therefore be considered that transcriptome data features an additional dimension, compared to conventional sequence-oriented data. The researcher’s interest is not limited to obtaining transcripts for specific genes of interest (although this approach is a common one). The researcher may also seek transcripts exhibiting an expression profile which indicates an association with the biological mechanism in question.

As a crude example, a cancer researcher might compare RNAseq data from cancerous and normal tissues, with the assumption that genes which are differentially expressed between these samples must have some functional relationship to the observed cancerous growth. The researcher might also narrow their search by focussing not on the entire transcriptome, but on a subset of gene families that are known to associate with the given phenotype. A combination of approaches may be utilized to filter down and locate the colloquial “needle in a haystack” among many thousands of transcripts. On reflection, the process of navigating these data could be greatly improved in both rigour and efficiency with appropriate software interfaces to enable such cross-referencing of dataset features.

In October 2020 we released a novel web service, CrustyBase.org [3], which aims to meet these needs in the crustacean research community. Molecular studies in these aquatic arthropods have surged in demand over the last two decades with the rise of the aquaculture industry, as farmers of valuable crabs, prawns and lobsters seek to improve production [4, 5]. Ecologists have also taken note of these animals as climate change disrupts crustacean zooplankton populations forming the basis of marine food webs [6, 7]. Their impact on marine ecosystems cannot be overstated, due to the critical role of crustacean zooplankton in marine food webs [6, 7]. Nonetheless, research in crustacean genomics is confined to a relatively small group of researchers around the world, making this an ideal field to test the novel, exploratory platform that we showcase here. The original CrustyBase release [3] allows users to search through available transcriptome datasets with a “Browser” feature, and then search for sequences in those datasets with an augmented BLAST (basic local alignment search tool). This tool runs just like a conventional BLAST search [8, 9], but dynamically renders transcript expression graphs and predicted protein domain models as part of the results view. This enables researchers to rapidly extract transcripts of interest and examine their structure and behaviour across the variety of experiments hosted on CrustyBase. These tools are complemented by a “Data import” flow which allows users to upload datasets of their own in the web interface with minimal effort. Uploaded datasets then go through an enrichment pipeline, where transcript quantitation metrics are computed and open reading frames are predicted. Protein sequences are then computed from open-reading frames and conserved domains in these proteins are predicted by searching against NCBI (National Center for Biotechnology Information) CD (conserved domain) database [10]. The enrichment pipeline is described in full in the original release article [3]. It is worth noting that the data flow featured in CrustyBase is tailored to transcriptome dataset. By cross-referencing gene expression and transcript sequence data (the two data types produced *en masse* by transcriptome studies), our platform allows researchers to efficiently leverage these data for meaningful biological insights.

The CrustyBase platform is part of an expanding community of crustacean genomics resources, with similar web platforms emerging in recent years. CrusTF [11] provides an interface for transcription factor gene data in a range of crustacean species. The Crustacean Annotated Transcriptome [12] database provides access to a curated set of annotated crustacean transcriptomes through a BLAST search feature and gene annotation search. Owing to improvements in sequencing technology, we have also seen the publication of genome assemblies for at least seven crustacean species [13–23].

### Construction and content

CrustyBase is built with the Django web framework for the Python programming language. The broad architecture of the application has proven stable and remains largely unchanged since initial release [3]. We have provided users with a service uptime over 99.5% (as reported by uptimerobot.com) with one significant event lasting 12 h due to infrastructure outage. During this time, we also migrated the web server to our infrastructure provider’s new server hardware with zero downtime. The new hardware provided by the Australian Research Data Commons (ARDC) Nectar Research Cloud should increase web server performance, resulting in faster processing of jobs and response to web requests. In the two years since release, user engagement with the platform has increased steadily. Before discussing new developments on the platform, we will first report some user statistics to briefly describe user engagement to date.

### Usage metrics

We utilized two different approaches to measuring uptake and impact of CrustyBase.org. Google Analytics provide broad demographic insights into web traffic on the site. Metrics calculated from a three-year period following release of the platform are displayed in Table 1. The first two rows show the country of origin and number of distinct users making requests to the web service reported by Google Analytics. A total of 22,469 web requests were made against CrustyBase.org, with the majority of users originating from Germany (1491), the USA (539), Australia (476) and China (371). However, out of 3413 users identified by Google analytics, we found that only 56 users have registered an account with the service, which enables them to save results and upload datasets.

**Table 1:** User metrics for CrustyBase.org based on Google Analytics reports and server log files from October 2020 – October 2023

Countries reached	74
Distinct users	3413
Registered users	56
BLAST search submissions	6651
Transcripts downloaded	11,463
Files downloaded	9676
Datasets uploaded	19
Total transcripts deposited	3,966,051
Total nucleotides deposited	3,855,038,533

It should be noted that Google Analytics reports are subject to limitations, particularly when users opt to disable tracking cookies or engage with multiple devices. These instances can significantly compromise the accuracy of user volume reporting. In association with web traffic reported by analytics, we can see that 6651 submissions were made to the BLAST tool (Table 1), which requires users to submit a nucleotide or protein sequence to search against a selection of transcriptome datasets. From the results of these BLAST searches, users identified and downloaded a total of 9676 files which included 11,463 individual transcripts. Our registered users uploaded a total of 22 new transcriptome datasets, bringing the current database content to 31 datasets. These datasets introduce new species including two copepods, four lobsters (homarid and achelatan), two crayfish and a freshwater prawn. Of particular note are the datasets which complement the original *Panulirus ornatus* library, providing a comprehensive account of this species across embryonic development, larval molt stages and adult tissue distributions.

### Bug fixes and enhancements

A significant number of bug fixes were developed and applied since the release of the platform, typically in response to automated error notifications generated by the web server. Most repairs were minor and were usually actioned and applied within 24 h of the notification. None of them impacted the integrity of data on the service. A full list of changes can be seen in supplementary file 1.

A small number of enhancements were also applied to the software to improve user experience and application performance. These enhancements are listed in supplementary file 2. The most notable improvement here was the implementation of automated database and filesystem backups to Amazon web services S3 storage. This happens every 24 h, with daily, weekly and monthly backups being archived in cloud storage. This greatly improves the resilience of the platform, since application state can be restored to a new instance of the service at any time, should disaster recovery be required.

### New features

In the original CrustyBase release, the only tool for finding and extracting transcripts within a dataset was the BLAST tool. While this tool is ubiquitous and highly effective, it is only of use when the researcher knows the approximate genetic sequence of the transcripts they seek. There are two further use cases that we have sought to address, which arise from the following situations:

1. The researcher knows the database identifier(s) for the transcript(s) that they seek.
2. The researcher is seeking transcript(s) associated with a given set of keywords.

The first situation is typical of users who are accessing the dataset externally to CrustyBase (e.g. on their local computer, or through the associated NCBI BioProject). We offer a simple “Extract” tool to resolve this situation. This tool takes a list of transcript IDs which can either be uploaded as a file or pasted directly into a text box. The listed IDs are then extracted from the selected dataset and associated gene expression and protein structure data are displayed to the user.

The second situation is more complex and might have numerous solutions. A conventional approach to this issue involves keyword search against a pre-computed annotation database, but this is not yet part of the CrustyBase enrichment pipeline. Instead, we chose to develop a solution that was suggested in the first release of the platform, which utilizes predicted protein domain data that is already generated in the enrichment pipeline [3]. This new tool is named “Domain search” and allows the user to enter a set of keywords and search the selected datasets for transcripts which are predicted to encode conserved protein domains related to the given keywords. This novel approach to mining transcriptome libraries enables the user to search broadly by gene function, rather than by canonical gene names, and in some cases may allow the extraction of entire gene families in one search.

### Extract tool

**Fig. 1:**
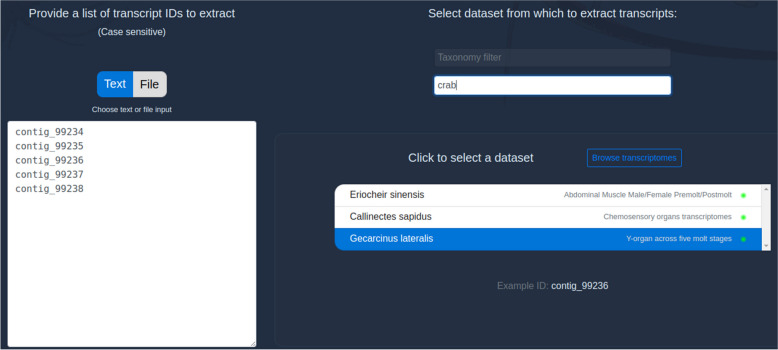
The input interface for the Extract tool allows users to select a dataset from which to extract a specific list of transcripts by identifier. The tool can be accessed at https://crustybase.org/extract

**Fig. 2:**
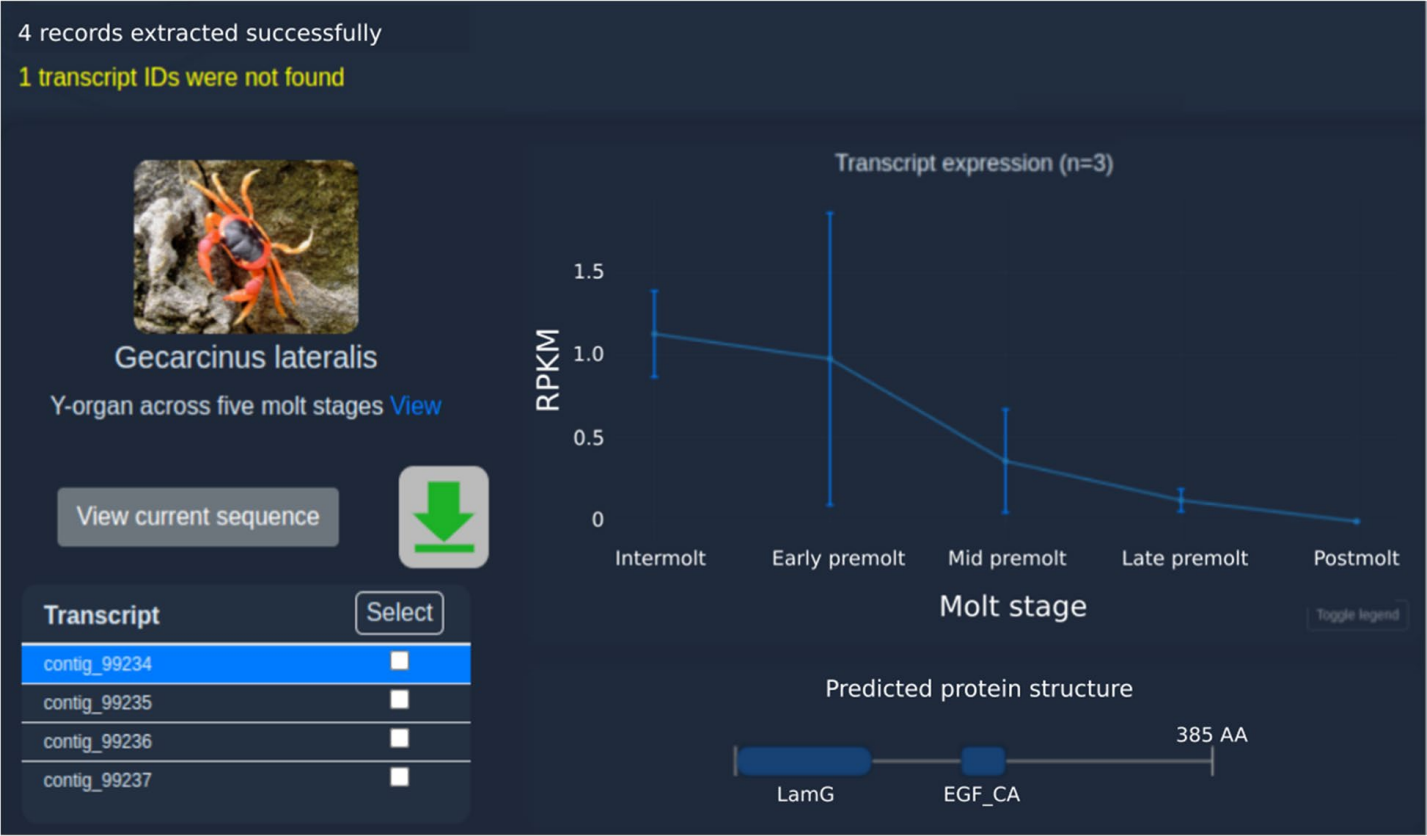
Result interface for the extract tool. The left side of the display shows the source dataset and provides navigation of transcript selection (table rows) and download options (checkboxes and green icon). The right side of the display provides interactive figures for gene expression and predicted protein structure of the selected transcript

**Fig. 3:**
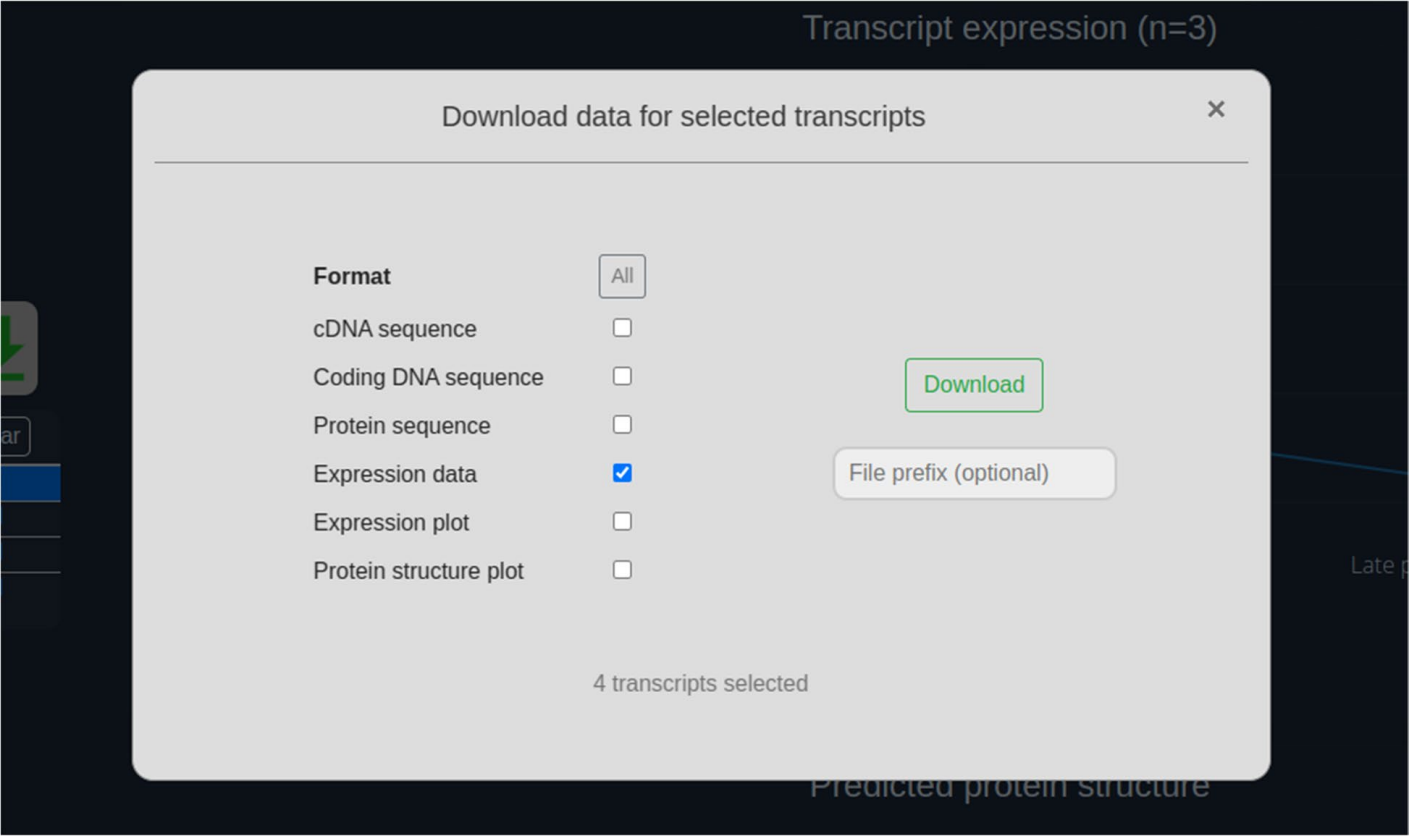
Dialog for downloading data for selected transcripts in the”Extract” tool results. Here the user has selected only the”Expression data” format to return a CSV-formatted dataframe of transcript abundance

The Extract tool is available at https://crustybase.org/extract. This tool takes advantage of existing BLAST indexes in the database, enabling instant extraction of transcripts with known identifiers. The user can provide a list of identifiers either by pasting directly into the webform (Fig. 1) or by uploading a text file containing one identifier per line. They then select the dataset they wish to extract these identifiers from. On selection, an example of the transcript ID format is shown below the dataset picker such that the user can confirm that their identifiers match the selected dataset before submitting the request. In the backend, the “blastdbcmd” command-line tool (part of the BLAST + package [9]) is used to extract records from the target BLAST database. Transcript identifiers which have been successfully extracted are then cross-referenced against expression and predicted domain records to return to the user for display. In the results interface (Fig. 2), we re-use components from the existing BLAST tool [3] to provide a familiar list of transcript identifiers, which can be clicked to display the associated data. We also provide a similar set of utilities to allow data downloads in the various formats offered by CrustyBase, which includes FASTA formatted sequences (cDNA, coding DNA and protein), transcript quantitation (CSV format) and static figures for transcript quantitation and protein domain prediction (PNG format) (Fig. 3). The user will also recognise the “Transcript expression” and “Predicted protein structure” plots from the BLAST tool. The interactive expression plot shows variation in transcript abundance across the experimental features, for which detailed descriptions are displayed or hidden with the "Toggle legend" button. The protein structure plot displays predicted protein domains as blue blocks whose names are highlighted for clarity on cursor hover. Clicking on a domain opens the associated profile page on the NCBI CD database, to offer more information to the user.

### Extract tool – case study

As a case study, we can assume that the user has obtained a set of transcripts from the NCBI BioProject (PRJNA413254) of the blackback land crab *Gecarcinus lateralis*. The user pastes the identifiers for five transcripts into the text input and selects the appropriate dataset before submitting their request (Fig. 1).

The extracted transcripts are returned to the user in less than a second (Fig. 2). Note that one transcript in the request was not found in the database – as shown in yellow text in the top-left corner. The user may then click through the transcript table (bottom-left) and study the associated data in the expression and protein structure panes (right). Alternatively, they may wish to download the quantitation data associated with these transcripts, in which case they could select all checkboxes in the transcript table and click the green “download” button to present the dialog shown in Fig. 3. Here they can select the “Expression data” format to download the required data.

### Domain search

The domain search tool is available at https://crustybase.org/domains. This tool takes advantage of conserved protein domains predictions which are computed as part of the data enrichment pipeline. These data are displayed in the BLAST and Extract tool results (see Fig. 2), but this tool searches directly against these annotations with keywords as an input. To allow text search against protein domains, it was necessary to build a library of text annotations for all protein domains in the CrustyBase database. This was achieved using the NCBI Entrez API [24] through the BioPython library [25], from which the “subtitle” and “description” fields are requested for each domain accession in the database. We refer to these data as “domain annotations”, since they include a structured description of each domain accession in the database (Table 2). When a new dataset is uploaded, CrustyBase will automatically call this API to fetch annotation data if novel domains are detected. Furthermore, the server runs a scheduled monthly update of protein domain descriptions to ensure that domain annotations are current.

**Table 2:** Example of domain annotation data retrieved from the Entrez Conserved Domain Database API for accession cl28897. The description field for this accession is 1926 characters in length but has been truncated here for brevity

Accession	Name	Subtitle	Description
cl28897	7tm_GPCRs	seven-transmembrane G protein-coupled receptor superfamily	This hierarchical evolutionary model represents the seven-transmembrane (7TM) receptors, often referred to as G protein-coupled receptors (GPCRs), which transmit physiological signals from the outside of the cell to the inside via G proteins…

With these domain annotations available to reference, it is relatively straightforward to build a keyword search interface for protein domains. We used Django’s built-in SearchQuery class to build a set of search vectors against these database fields, enabling efficient full-text search on the PostgreSQL database. This search function typically returns a list of matching domain accessions in under two seconds. This list of domains is then cross-referenced against datasets to identify transcriptomes encoding the matching domains. A second search is then made on selection of a dataset to fetch the domains, transcripts and transcript data associated with that dataset. This second search typically takes 10 s but could be dramatically improved with a pre-built search index (described later in “Priorities for the next major release”).

**Fig. 4:**
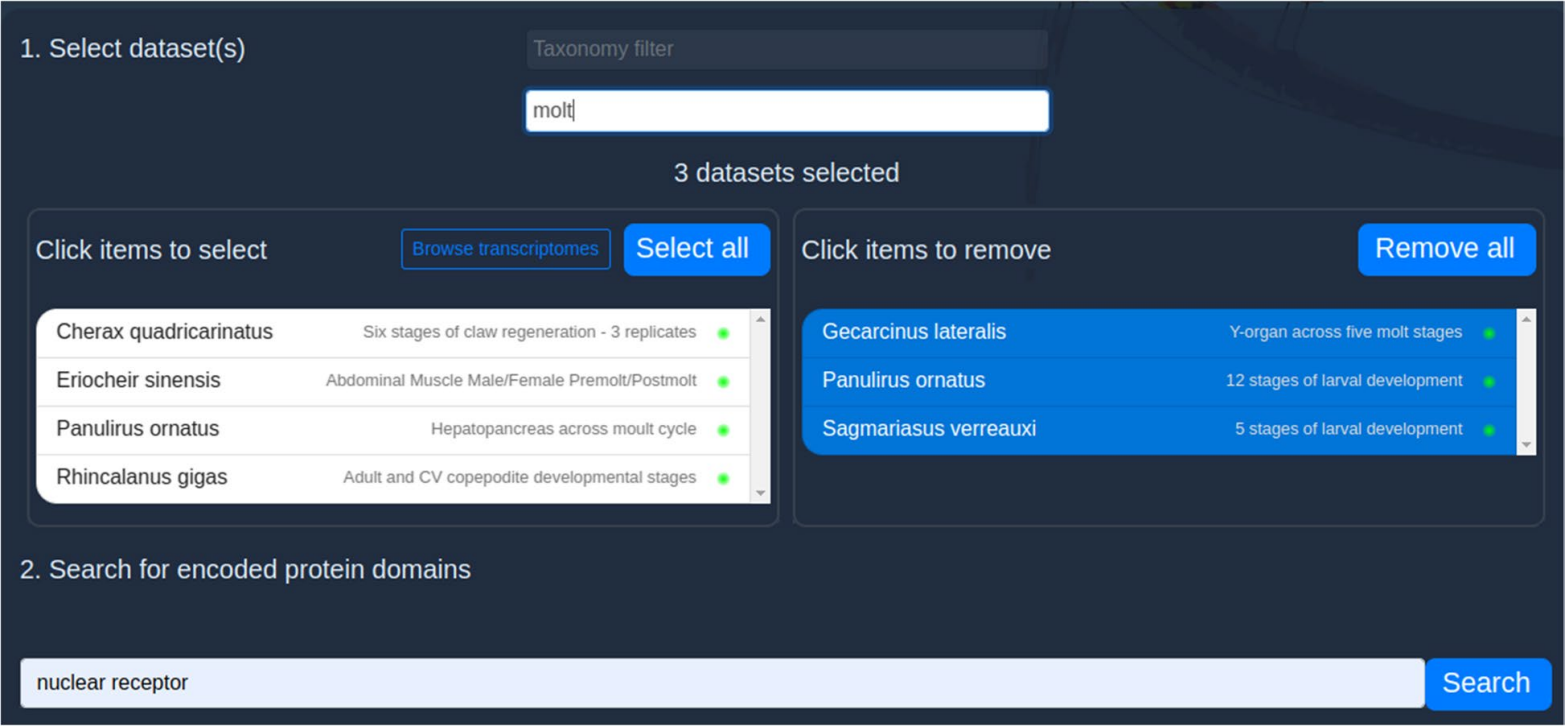
User interface for the”Domain Search” tool. Datasets are picked in the upper panel (1) before a keyword search can be made in the lower panel (2)

How to present this feature to the user presented more of a challenge. There is no use in returning tens of thousands of transcripts across all available datasets which match the user’s search term. We instead sought to provide the user with a fine-grained mechanism to explore these results in an intuitive and transparent way. To begin with, the user must select the dataset(s) that they are interested in searching; we assume that people come to CrustyBase in pursuit of some biological insight that is represented only in certain datasets. The dataset picker (Fig. 4) input is identical to that found in the existing BLAST tool interface [3], allowing the user to search by taxonomy and keywords for datasets of interest. The user can then enter keywords targeting transcripts of interest into a simple text input and submits the search request.

**Fig. 5:**
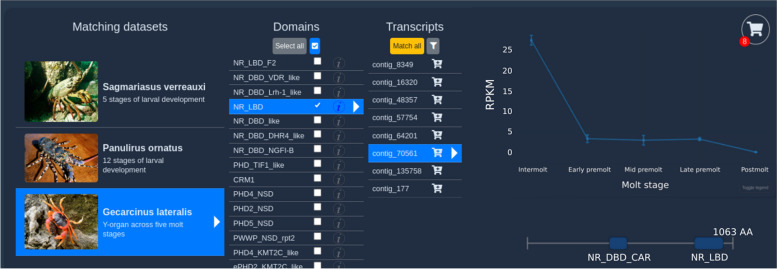
Results interface for the”Domain search” tool. Interaction with this drilldown flows from left to right, from dataset through to transcript. The two plots on the right display expression and protein domain structure for the selected transcript. The cart icon in the top right shows the number of transcripts selected for export

Since the search is limited to specific datasets, search time and result complexity are reduced from the beginning. We designed the results interface to display results as a drilldown (similar to a file explorer), with datasets as the root (Fig. 5). The user selects one of the available datasets, which loads a list of matching protein domains encoded by that dataset’s transcriptome. The user can then select one or more of these protein domains, which triggers the display of the transcripts encoding those domains. Selecting a transcript then triggers the display of protein structure and expression plots for that transcript. This entire process occurs asynchronously within a single webpage to maximise clarity for the user. After the results have been retrieved, navigating to the transcript level typically requires less than 15 s, as assessed against the proficiency of a seasoned user of the platform.

There are two options for cross-referencing selected domains against transcripts, which come into play when more than one domain is selected. The default option (“Any” in the user interface) uses OR logic when cross-referencing against transcripts. That is, it will display transcripts matching *any* of the selected domains. The alterative option (“All” in the user interface) uses AND logic, such that transcripts will be displayed only if they encode *all* the selected domains. The latter presents the user with far fewer (often zero) transcripts but allows the user to search for combination domain architectures. To further assist the user in navigating the transcripts returned (of which there can be many thousands), we include a feature which allows the user to filter transcripts based on transcript quantitation and predicted protein length. Some datasets include many transcripts for which no quantitation data is available; the first filter enables the user to exclude these transcripts from the display. The second filter allows the user to express a minimum predicted protein length (in amino acids) for transcripts to display.

**Fig. 6:**
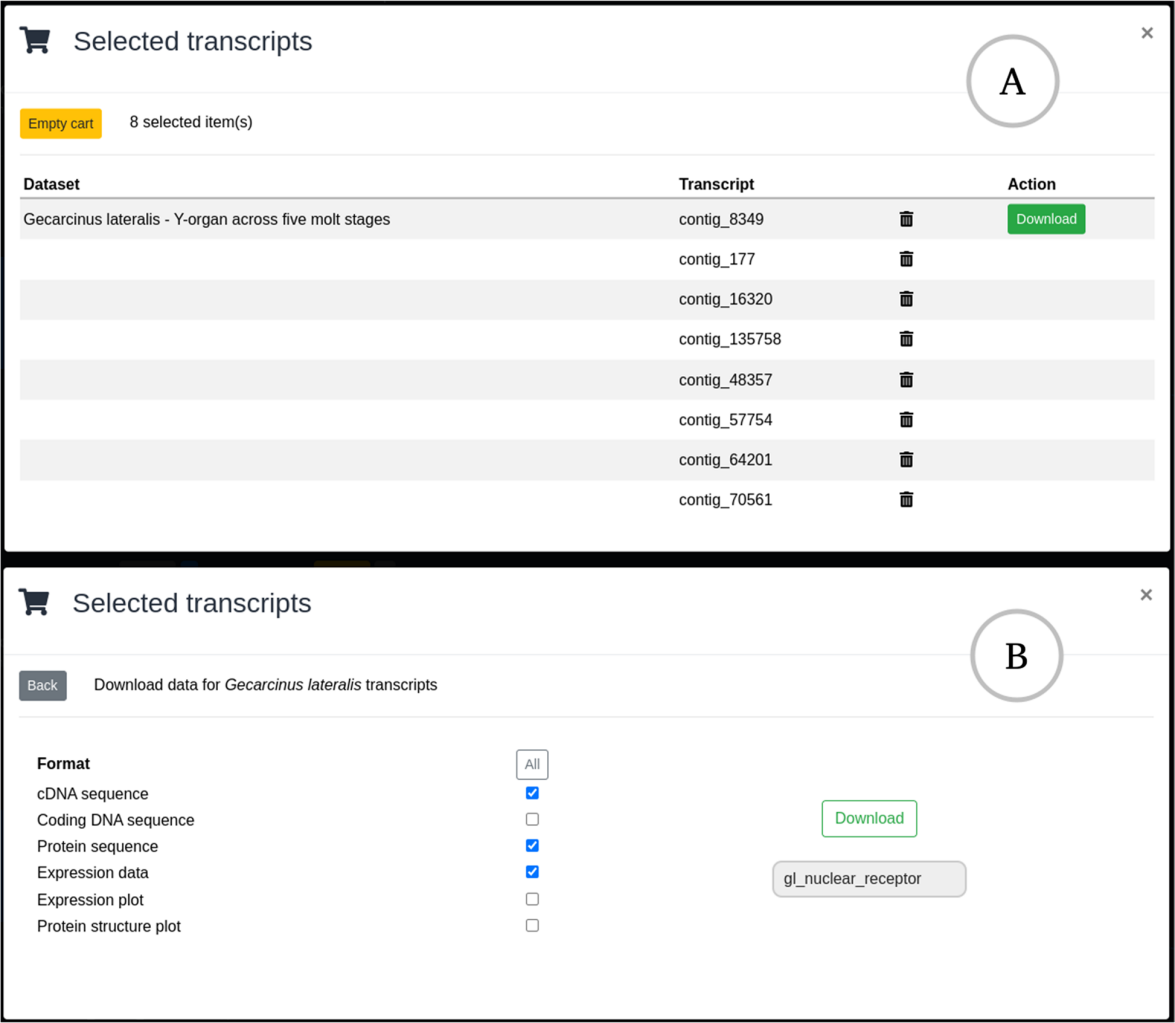
Checkout dialog for downloading selected transcripts. Panel **A** shows the initial dialog which show selected transcripts grouped by dataset. Each dataset has a separate button to download and allows users to clear unwanted transcript selections before download. Panel **B** shows the download dialog where the user can select the required datatypes and enter a filename for the downloaded archive

When the user identifies transcripts of interest, they can take advantage of the “cart/checkout” feature that has been developed as part of this tool. Each transcript row has a cart icon (Fig. 5) that will add that transcript to the “cart”. In the top-right corner, a cart icon appears with a count of the number of transcripts that have been added. When the user is satisfied with the transcripts that they have gathered, they can click on this icon to “go to checkout” and download a selection of data types related to the selected transcripts (Fig. 6).

### Domain search case study – gene family

To demonstrate the utility of the Domain Search tool, we will assume that a researcher wishes to investigate the role of nuclear receptor genes in crustacean molting. First, they enter the term “molt” into the keyword search field and pick three datasets that represent the appropriate biological context (Fig. 4). They then enter the term “nuclear receptor” into the domain search field and click “search”.

They are soon presented with a list of “Matching datasets”. They select the *Gecarcinus lateralis* dataset, which features Y-organ samples across five stages of the molt cycle. This prompts the associated protein domain and transcript data to be fetched from the database; available protein domains are now displayed in a second column. The user then selects the “NR_LBD”, which, after hovering on the information icon, can be seen to encode the “nuclear receptor ligand-binding domain”. This triggers the immediate display of 35 transcripts which are predicted to encode this domain. The researcher clicks through a few of these transcripts and realises that most of these proteins are too short to be of interest to them. They open the transcript filter and set the minimum protein length to 600 amino acids. This immediately reduces the list to eight transcripts, all of which display an expression pattern that appears to change significantly across the molt cycle (Fig. 5).

The user then adds all these transcripts to the cart and brings up the checkout dialog by clicking on the cart icon (Fig. 6; panel A). They can then review the selected transcripts before moving to the download dialog for their selected dataset (Fig. 6; panel B). They select cDNA and protein sequences, as well as raw expression data for these transcripts before downloading these data for further analysis. In approximately five minutes, the researcher has been able to navigate the entire CrustyBase database, narrowing their search down to eight transcripts of interest. With the raw data downloaded they can validate and explore these data to address the research question.

### Domain search case study – single gene candidate

**Fig. 7:**
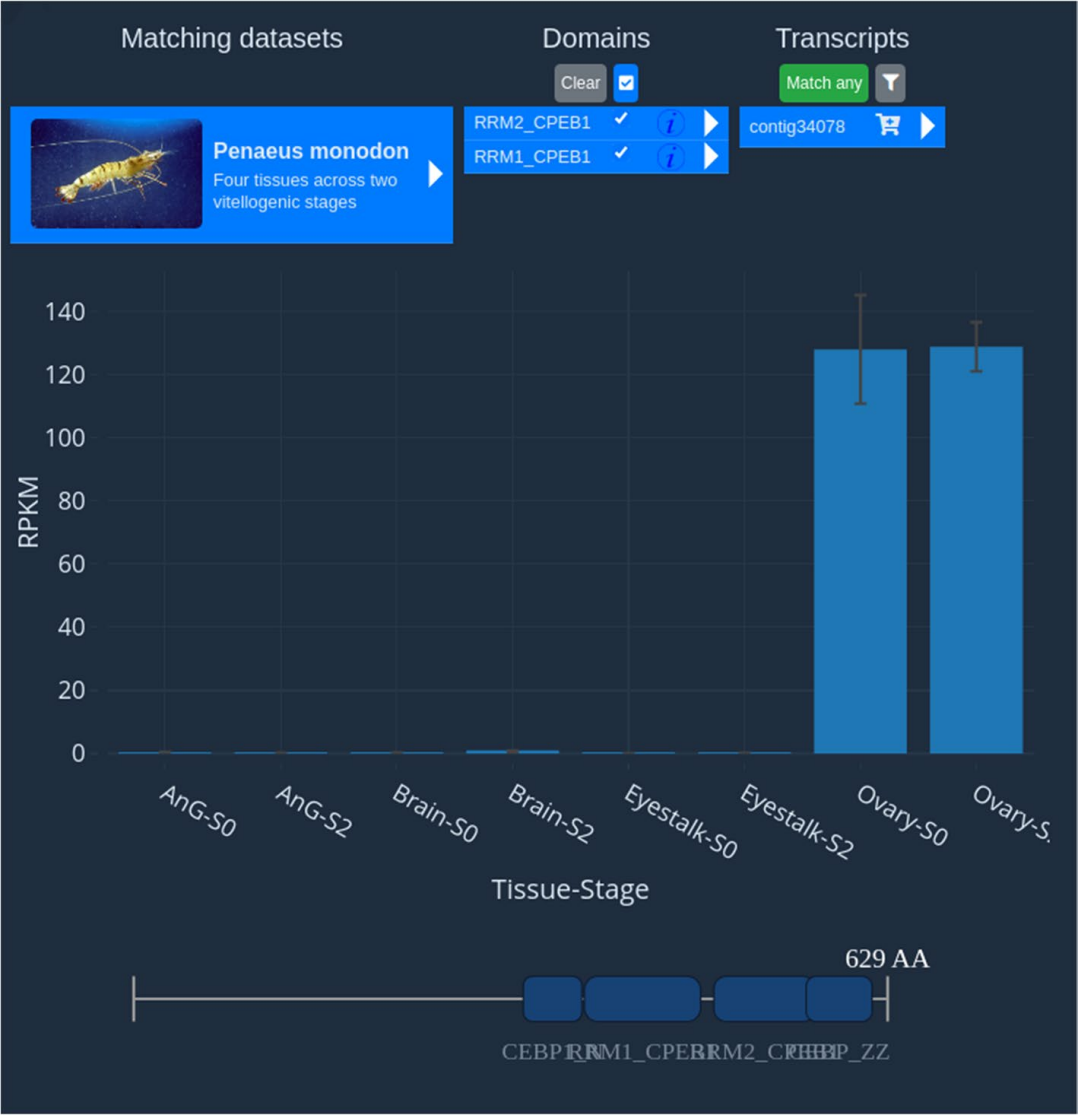
Isolation of an ortholog of Human *cpeb1* gene in Penaeus monodon with the Domain Search tool. The X-axis for this dataset shows four tissues (androgenic gland, brain, eyestalk and ovary) at two stages of reproductive maturity (previtellogenic S0 and vitellogenic S2). Interrogation of this dataset quickly confirms ovary-specific expression of the *P. monodon cpeb1* ortholog

In a second example, we will briefly investigate orthologs of the Human *cpeb1* gene, whose expression is known to be localized to ovary tissues in human studies. In this case, the researcher enters a direct search query for their candidate “cpeb1” and selects the *Penaeus monodon* dataset (https://crustybase.org/browser/?exp_id=agIKWkLzq3IB49SMor6zgwxuo), which was identified as relevant by searching for “ovary tissues” in the Browser tool. The Domain search tool locates two protein domains in this dataset—“RRM2_CPEB1” and “RRM2_CPEB2” which occur in just a single transcript. On clicking this transcript, the researcher immediately confirms that this ortholog of human *cpeb1* is highly expressed in the ovary (mean 128 RPKM) and virtually absent in eyestalk, androgenic gland and brain tissues (mean < 1 RPKM) (Fig. 7). This exemplifies the potential of the platform for rapidly collecting evidence to address nuanced biological inquiries.

### Future directions

After the initial release, we found it valuable to set clear objectives and communicate our intentions for the platform with the community. Here we have again attempted to distil our aims for further development, with objectives for the near future (within the next year) and in the long-term (beyond a year).

Of particular importance is the data processing pipeline that formats and enriches datasets uploaded by our users. Currently this process is coordinated by a series of Python scripts on an institutional HPC server. Should access to this resource be withdrawn, CrustyBase would lose the ability to import new datasets. We have therefore identified a high-priority activity to containerize this enrichment pipeline (e.g. Docker), such that it can be migrated easily between hosts. Another issue that has been raised is the omission of access to expression replicate data. Currently users can only visualize and download mean and standard deviation data, but the raw replicate data should be made available to facilitate downstream applications such as differential expression analysis. Finally, some of the power users of CrustyBase have requested that programmatic access to platform resources would accelerate research for those looking to access many thousands of records. To this end we would seek to implement a public web API for selected areas of the application, with user-facing documentation describing the available endpoints.

Looking further into the future, we would like to broaden the types of data hosted by CrustyBase. An obvious addition would be implementing annotation of transcripts as part of the data enrichment pipeline. This would give rise to a rather simple feature of searching directly against annotations to identify transcripts of interest. Implementing this has always been restricted by the compute required for BLAST annotation at the transcriptome scale, which would most likely be resolved by improved infrastructure. Another feature that is restricted by computing resources is the ability to upload sequencing reads for assembly and mapping. This would open the door to a plethora of datasets that are currently available in the NCBI Sequencing Read Archive but for which public assemblies are not available. We are also interested in how genomic data might be leveraged by the platform. Historically this has been of limited interest given the lack of available genomes for crustacean species, but improvements in sequencing technology will likely see high-resolution genomes for these species in the coming years. In the coming decade, we anticipate the advent of single-cell RNA-seq datasets for crustacean species, presenting a unique opportunity for the refinement of analytical capabilities within platforms like CrustyBase. Realizing this potential will necessitate the creation of novel database tables and datatypes in the application backend. Additionally, interactive user interfaces in the frontend will be instrumental in facilitating seamless data visualization for users engaging with these emerging datasets.

In addition to expanding the types of data that are exposed through the platform, we would also like to consider opportunities to collaborate and integrate with other public resources. CrusTome [13] is a collection of over 200 crustacean transcriptome assemblies which have been generated with a stringent pipeline to reduce redundancy. The consistency in quality across such a broad range of taxa makes this is an inscrutable resource for phylogenetic analysis. However, access to CrusTome is limited to downloading the data files for local analysis. By reusing components that exist already in CrustyBase, it would be possible to incorporate these data into a new “sequence curation” tool in our platform, thereby increasing the accessibility of this valuable resource.

### Priorities for the next major release of CrustyBase

1. Improve computational performance of domain search
2. Containerize import pipeline for reproducibility and increase resilience/mobility
3. Web API to enable programmatic access to data
4. Expose raw expression data for display and download

### Proposed long-term additions and improvements

1. Cross-reference against genomic data as this become more available for crustacean species
2. Enrichment pipeline upgraded to include BLAST annotation of transcripts
3. Transcriptome assembly and quantitation import pipeline to allow direct import of clean sequencing reads
4. Single-cell RNA-seq analysis capabilities

## Conclusion

We have reported here a series of enhancements, feature additions and corrections to the CrustyBase application, with the intention to improve transcriptome data accessibility for researchers of crustacean genomics. User metrics indicate that this modest community of researchers has found value in the resource, which in turn has motivated maintenance and development of the application. The two features additions that we report here offer two new means of accessing data from the transcriptome libraries on the platform. In particular, we hope that Domain search will allow our users to rapidly curate functional gene families and shed light on their activity across a variety of experiments. In the future, we are interested in exploring and developing additional interfaces to transcriptome data which might aid researchers in realising the full value of these datasets.

Designing interfaces to such complex data is very challenging, however. As domain experts in crustacean transcriptomics, we at least have a moderate insight into what, as researchers, we would like to do with our data. And yet, engagement with the wider community is critical to ensure that the interfaces we develop are of general use. We therefore welcome our users, and indeed readers of this article, to share any feedback that might improve the general utility of this platform for the community at https://crustybase.org/feedback.

Aside from serving a purpose in this specific subdomain of genomics, we hope that the platform might also demonstrate the potential to improve our interface to these complex and critical data. Every year, many millions of dollars are awarded globally to projects generating public transcriptome data, most notably for the purpose of biomedical research. Investing in the accessibility of these data is critical to realising their value. Public data which are effectively exposed to the community will be actively mined and scrutinized, gleaning all possible scientific value from it. Aside from enabling the consumption of these data, platforms also have an untapped potential for improving collaboration and peer-review. In each of these cases, the impact against the data made by the user will be directly proportional to the accessibility of that data. Therefore, we encourage directors of research funding to consider these platforms as a high impact research output, truly worthy of investment.

### Supplementary Information

**Additional file 1.** Bug fixes listed by date applied since public release of CrustyBase in October 2020.

**Additional file 2. **Minor enhancements to the service, listed by date applied since release of CrustyBase in October 2020.

## Data Availability

For security reasons, we do not intend to open-source the CrustyBase application code, but we encourage anyone wishing to collaborate on this project to contact us for access. Most datasets on CrustyBase are open access and available for anyone to search on the website. Many datasets include a link to an NCBI BioProject where the raw data can be downloaded. Where this does not apply, we suggest you contact the dataset owner directly to seek collaboration.

## Abbreviations

BLAST: Basic local alignment search tool
NCBI: National center for biotechnology information
