# Supplementary material for: CrustyBase v.2.0: new features and enhanced utilities to support open science
Source: BMC Genomics. 2024 Jan 29;25:121. doi: 10.1186/s12864-024-10033-7 (PMC10823621; doi:10.1186/s12864-024-10033-7)
Supplement: Supplementary file 1 — Additional file 1. Bug fixes listed by date applied since public release of CrustyBase in October 2020. [file 12864_2024_10033_MOESM1_ESM.docx]

# Bug fixes listed by date applied since public release of CrustyBase in October 2020.

| 18/01/23 | Fix BLAST results expanded view on small screens |
| --- | --- |
| 18/11/22 | Fix taxon query selection dropdown |
| 21/04/22 | Handle missing sequences without raising an error when extracting sequence from BLAST databases. |
| 10/12/21 | Fix a potential bug in rendering domain plot download. |
| 25/11/21 | Upgrade to BLAST v2.11.0+ |
| 15/09/21 | Fix call for queue position. |
| 31/08/21 | Fix downloads and domain plot when no protein sequence is available. |
| 14/08/21 | Fix rendering of scale bar in BLAST alignment for very short sequences. |
| 09/08/21 | Return a 404 error on BLAST search requests for datasets that don’t exist. |
| 01/08/21 | Apply a patch for errors with capitalized identifiers in the BLAST+ makeblastdb tool. |
| 31/07/21 | Limit the maximum number of hits in BLAST results to 20 to prevent poor performance on large results. |
| 31/07/21 | Update formatting of sequence identifiers in BLAST database to include dataset identifier. |
| 31/07/21 | Fix registration form validation messages. |
| 20/07/21 | Enforce lowercase usernames at registration and login. |
| 26/02/21 | Fix function for generating the URL for a species image. |
| 31/01/21 | Sanitize FASTA query title when building download file name. |
| 27/01/21 | Fix validation of BLAST form fields and improve form feedback on user error. |
| 10/11/20 | Add maximum length restriction to download file name prefix to prevent errors. |
| 10/11/20 | Truncate FASTA sequence titles to 50 characters to prevent violation of database field length constraint when saving BLAST results. |
