# Supplementary material for: CrustyBase v.2.0: new features and enhanced utilities to support open science
Source: BMC Genomics. 2024 Jan 29;25:121. doi: 10.1186/s12864-024-10033-7 (PMC10823621; doi:10.1186/s12864-024-10033-7)
Supplement: Supplementary file 2 — Additional file 2. Minor enhancements to the service, listed by date applied since release of CrustyBase in October 2020. [file 12864_2024_10033_MOESM2_ESM.docx]

# Minor enhancements to the service, listed by date applied since release of CrustyBase in October 2020.

| 03/01/23 | Upgrade Django to version 4.1.3 |
| --- | --- |
| 03/01/23 | Upgrade database to PostgreSQL 15 |
| 21/04/22 | Display selected BLAST hit sequence as a popup in the browser. |
| 06/09/21 | Implement automated database backups. |
| 23/08/21 | Restructure BLAST working directory. |
| 09/05/21 | Improve display of BLAST results on smaller screen size. |
| 08/05/21 | Add popup legend for x-axis labels in BLAST result expression graph. |
| 08/05/21 | Add announcements panel to the home page. |
| 28/03/21 | Add “maintenance mode” view for downtime during planned maintenance. |
